# Association between latent classes of tobacco, e-cigarette, alcohol, and illicit drug use patterns and depression among Thai adolescents

**DOI:** 10.1016/j.abrep.2026.100738

**Published:** 2026-08-26

**Authors:** Renarebecca Sagayanathan, Wit Wichaidit, Martin Stafström

**Affiliations:** a Faculty of Medicine, Lund University, Sweden; b Department of Epidemiology, Faculty of Medicine, Prince Songkla University, Thailand; c Division of Social Medicine and Global Health, Faculty of Medicine, Lund University, Sweden

**Keywords:** Adolescent mental health, Polysubstance use, Alcohol and tobacco use, Gender identity, Thailand

## Abstract

**Background:**

Adolescent substance use behaviors often co-occur and are associated with poorer mental health. While latent class analysis (LCA) has identified adolescent substance use patterns in high-income countries, evidence from Southeast Asia remains limited.

**Aim:**

To identify gender-stratified substance use patterns among Thai adolescents aged 16–17 years and examine their associations with elevated depressive symptoms.

**Methods:**

We conducted a cross-sectional analysis of **9188** Thai adolescents aged 16–17 years using data from the 5th Thai National School Survey on Alcohol Consumption, Substance Use and Other Health-Risk Behaviors. Fourteen indicators of lifetime and recent tobacco, e-cigarette, alcohol, and illicit substance use were included in gender-stratified LCA models. Elevated depressive symptoms were assessed using the Patient Health Questionnaire-2 (PHQ-2; score ≥ 2). Logistic regression estimated associations between latent class membership and elevated depressive symptoms after adjustment for sociodemographic, academic, and lifestyle covariates.

**Results:**

Four latent classes were identified among males and three among females and gender-diverse adolescents. Compared with the minimal/non-use class, all substance-using classes showed higher odds of elevated depressive symptoms. The highest odds were generally observed in the high polysubstance-use classes. Among males, the experimental cannabis/kratom and tobacco-related use class showed odds comparable to the high polysubstance-use class.

**Conclusions:**

Distinct gender-stratified substance use patterns were associated with elevated depressive symptoms among Thai adolescents. These findings extend evidence on adolescent substance use heterogeneity in Southeast Asia and support the use of latent class analysis to identify adolescents at greater mental health risk. Longitudinal studies are needed to clarify temporal relationships.

## Highlights

• Gender-stratified latent class analysis identified distinct substance use patterns among Thai adolescents.
• Polysubstance showed higher odds for elevated depressive symptoms.
• In males, the cannabis/kratom and tobacco-related class had odds comparable to the polysubstance class.
• Substance use patterns and depressive symptom risk differed by gender and latent class.
• Findings highlight heterogeneity in adolescent substance use and mental health vulnerability.

## 1 Introduction

Globally, approximately 1 in 7 (14%) adolescents experience mental health conditions, representing nearly 166 million young people worldwide, with anxiety and depression accounting for a substantial proportion of this burden (UNICEF, 2021). Adolescent depressive symptoms are multifactorial and have consistently been associated with substance use behaviors (Danzo et al., 2017; Xu et al., 2016). Importantly, adolescent risk behaviors rarely occur in isolation. Rather, behaviors such as smoking, alcohol consumption, illicit drug use, and other health-risk behaviors tend to cluster together, reflecting shared psychological, social, and disinhibitory pathways (Akasaki et al., 2019). Evidence also suggests a bidirectional relationship between depressive symptoms and substance use, whereby adolescents may engage in substance use as a coping strategy, while substance use itself may co-occur with worsening mental health symptoms over time (Danzo et al., 2017).

These distinct patterns of substance use may reflect differing levels of psychosocial and neurobehavioral vulnerability among adolescents. Prior research suggests that adolescents who engage in polysubstance use are more likely to transition toward broader substance involvement over time and exhibit poorer psychosocial and mental health outcomes compared with single-substance users (Akasaki et al., 2019; Bell, 2016; Nath et al., 2022; Walinga & Stangor, 2014). Transitional studies further indicate that some adolescents progress along a continuum of substance use across adolescence and young adulthood (Dennermalm et al., 2023; Merrin et al., 2018). Such progression may be influenced by underlying neurodevelopmental vulnerabilities and behavioral dysregulation during critical periods of brain maturation (Conrod & Nikolaou, 2016; Volkow et al., 2019). Consequently, latent classes may capture broader underlying vulnerability profiles that are not adequately reflected when substances are examined independently. A latent class approach may therefore provide a more informative framework for understanding co-occurring substance use behaviors and their relationship with depressive symptoms among adolescents.

Most research examining adolescent substance use patterns has been conducted in high-income countries and has consistently identified several overarching latent class structures, including non-use or low-use classes, single- or dual-substance use classes, moderate polysubstance use classes, and high polysubstance use classes (Halladay et al., 2020; Marshall, 2014). Across studies, the most common substance combinations include alcohol only, tobacco and alcohol, cannabis and alcohol, or broader polysubstance involvement incorporating tobacco, alcohol, cannabis, and other illicit substances. While these broad typologies appear relatively consistent across settings, variations in study populations, developmental stage, substance initiation patterns, and measurement timeframes limit direct comparability between studies (Halladay et al., 2020). Importantly, evidence from Southeast Asia remains limited despite substantial cultural, regulatory, and socioeconomic differences that may influence adolescent substance use behaviors and associated mental health outcomes.

Thailand offers a particularly important context for studying adolescent substance use and mental health. Adolescents aged 10–19 years comprise approximately 11% of Thailand's population (UNFPA, 2023). Thailand ranks second after the Philippines for adolescent alcohol use within the ASEAN region (Pengpid & Peltzer, 2015), and the World Health Organization has identified alcohol and substance use as major adolescent health concerns in Thailand (WHO, 2022). In 2019, approximately 2.7 million Thai youths reported drug use and 2.5 million were identified as new alcohol users, while nearly all adolescents under the legal purchasing age were reportedly still able to purchase alcohol (Siamrath, 2019). Substance use patterns also vary substantially by gender, with males more commonly reporting cannabis, kratom, and methamphetamine use, while females more commonly report hypnotics, cough syrup, and anxiolytic use (ibid). Older adolescents aged 16–17 years represent a particularly important developmental group because this period is associated with increasing autonomy, academic pressure, peer influence, and greater experimentation with substance use behaviors. Despite these concerns, evidence on adolescent substance use patterns and mental health in Thailand remains limited, with only a small number of prior latent class studies conducted in the region (Assanangkornchai et al., 2018; Park & Kim, 2017).

To our knowledge, no large school-based study in Thailand has characterized gender-stratified latent classes of tobacco, e-cigarette, alcohol, and illicit substance use and examined their association with depressive symptoms among older adolescents. Therefore, the objectives of this study were: (1) to identify latent classes of substance use behaviors among Thai adolescents aged 16–17 years, and (2) to examine associations between these latent classes and screening positive for elevated depressive symptoms. Based on prior literature, we hypothesized that adolescents belonging to polysubstance use classes would demonstrate higher odds of screening positive for elevated depressive symptoms compared with non-users, with potential gender differences in these associations. Several sociodemographic and psychosocial factors have previously been associated with adolescent substance use and depressive symptoms and were therefore included as covariates in the adjusted analyses.

## 2 Methods

### 2.1 Study design and population

This population-based cross-sectional study used data from the 5th National School Survey on Alcohol Consumption, Substance Use, and Other Health-Risk Behaviors in Thai adolescents, conducted between November 2020 and March 2021. The survey assessed tobacco, e-cigarette, alcohol, and illicit substance use, consumption patterns, and health-risk trends. Schools were randomly selected within provinces with the intention of representing rural, and urban settings across all the 12 educational regions of Thailand, resulting in a sample of 113 schools in 21 provinces and one Bangkok district. Responses were voluntary, self-administered, and anonymous.

A stratified multistage cluster sampling design was used. First, 21 provinces and one district of Bangkok were randomly selected from Thailand's educational regions. Within each selected province, schools were stratified by type (public urban secondary schools, public rural secondary schools, private secondary schools, vocational commercial schools, and vocational technical schools), and one school from each available stratum was randomly selected. Some provinces did not contain all school types, resulting in fewer than five schools being sampled in those provinces. In total, 113 schools participated.

Within each selected school, classrooms were randomly sampled from eligible grade levels. Three classrooms were selected from Year 11 where available, or all classrooms were included when fewer classrooms existed. All students within sampled classrooms were invited to participate. Because selection probabilities differed across provinces and school types, analyses accounted for clustering and unequal sampling weights. Responses were voluntary, self-administered, and anonymous. In total, the study included 24,143 students from general education (Years 7, 9, 11) and vocational programs (Vocational Certificate Year 2), aged 12–19 years.

For the present analysis, we focused on students aged 16–17 years (students in Year 11) because late adolescence is associated with increased academic pressure, greater social autonomy, and elevated vulnerability to both substance use and depressive symptoms. The final sample comprised 9188 students, including 4880 females (55%), 3552 males (40%), and 404 (5%) gender-diverse students, with an overall response rate of 98%. Incomplete responses (<70% answered) were excluded. Restricting the sample to older adolescents improved developmental homogeneity and reduced potential age-related confounding.

### 2.2 Study instrument and data collection

Data were collected using a self-administered questionnaire on alcohol, substance use, and health-risk behaviors. Verbal informed consent was obtained by trained staff. Students received a brief introduction and explanation before completing the questionnaire in class and submitting it in sealed envelopes. Privacy and confidentiality were maintained throughout.

### 2.3 Measurement of depressive symptoms (PHQ-2 ≥ 2 points)

Depressive symptoms were assessed using the Patient Health Questionnaire-2 (PHQ-2), a brief screening instrument commonly used in large epidemiological studies to identify elevated depressive symptomatology rather than establish clinical diagnoses. Adolescents reported the frequency over the preceding two weeks of: 1) having little interest or pleasure in doing things and 2) feeling down, depressed, or hopeless. Response options ranged from 0 (“not at all”) to 3 (“nearly every day”). Combined scores of 0–1 were categorized as negative for elevated depressive symptoms, while scores of 2–6 were categorized as screening positive for elevated depressive symptoms. A lower cutoff of ≥2 was selected based on meta-analytic evidence supporting improved sensitivity for population-based screening contexts, particularly when the objective is to identify adolescents with poorer mental health rather than diagnose depressive disorders (Levis et al., 2020; Manea et al., 2016).

### 2.4 Measurement of substance use behaviors

Substance use was assessed using self-reported measures of tobacco, e-cigarette, alcohol, and illicit drug use across lifetime, past 12 months, and past 30 days. For the present latent class analysis, only lifetime and past-30-day indicators were retained to distinguish experimentation from more recent or recurrent use patterns while minimizing extended recall periods.

Students reported whether they had used cigarettes, e-cigarettes, alcohol, and illicit substances including cannabis, kratom-containing preparations (4 × 100), methamphetamine, inhalants, cough syrup misuse, sedatives, and other non-medical psychoactive substances. Anxiolytic, antihistamine, and cough syrup use was specified for non-medicinal purposes.

Frequent use of cigarettes and e-cigarettes during the past 30 days was defined as ≥20 days of use. Risky alcohol consumption was assessed using the AUDIT-C based on drinking frequency, quantity, and binge drinking (Bradley et al., 2007). Cutoffs were ≥ 4 points for males and ≥ 3 points for females and gender-diverse adolescents (Flentje et al., 2020; Liskola et al., 2018). All indicators were dichotomized for analysis. Non-response for substance indicators was infrequent and coded conservatively as non-use.

### 2.5 Statistical analyses

Analyses were performed using STATA version 18. Frequencies of lifetime and past-30-day substance use indicators were calculated prior to latent class analysis (LCA) (Sinha et al., 2021). Fourteen indicators representing tobacco, e-cigarette, alcohol, and illicit substance use patterns were included in the LCA models. Latent class analyses were conducted separately by gender identity to account for potential heterogeneity in substance use patterns across groups.

Indicators included lifetime smoking, frequent smoking (past 30 days), lifetime e-cigarette use, frequent e-cigarette use, lifetime alcohol use, risky drinking (AUDIT-C), lifetime and past-month use of cannabis/kratom-related substances, stimulant/opioid substances, club/party drugs, and non-medical pharmaceutical use. Models were estimated using STATA gsem.

Class solutions were evaluated using Akaike Information Criterion (AIC), Bayesian Information Criterion (BIC), log-likelihood, and entropy (≥0.8 indicates clear classification). Parsimony and interpretability guided final selection. Additional classes were retained only when they contributed meaningful differentiation of substance use patterns. Posterior probabilities were computed to assign participants to their most likely latent class membership (Weller et al., 2020). Models were estimated using multiple iterative solutions and compared for stability and interpretability. However, formal multiple random-start procedures were not implemented.

Descriptive statistics were examined across latent classes and covariates associated with screening positive for elevated depressive symptoms. Logistic regression models estimated associations between latent class membership and elevated depressive symptoms, using the minimal/non-use class as the reference category. Covariates were selected a priori based on prior literature demonstrating associations with adolescent substance use and depressive symptoms and included gender identity, GPA, grade change, weekly allowance, residence, and sleep duration.

Missing data for covariates and depressive symptom measures were excluded using complete-case analysis. Among students completing the survey, missingness for these variables was below 5%, respectively. The characteristics of non-responders did not differ from responders. Results are presented as adjusted odds ratios (aORs) with 95% confidence intervals and statistical significance set at *p* < 0.05.

### 2.6 Ethical considerations

The study was approved by the Human Research Ethics Committee, Faculty of Medicine, Prince of Songkla University (REC.63–446–18-2). Verbal informed consent was obtained, and confidentiality was maintained. A waiver of written consent was granted due to the sensitive nature of the questions.

## 3 Results

The descriptive statistics table (Table 1) provides gender-stratified distributions of the fourteen variables used in the LCA model, based on lifetime and frequent use in the past thirty days for each of the classifications of substance used. (See Table 2.)

**Table 1:** Descriptive statistics of indicator variables used in the Latent Class Analysis (LCA) model to characterize the user pattern of substance use among study participants aged 16–17 from the 5th National School Survey on Alcohol Consumption, Substance Use and Other Health-Risk Behaviors in Thai Male, Female and Gender Diverse adolescents respectively in 2020/2021.

	LIFETIME EXPOSURE		FREQUENT EXPOSURE⁎ (Past 30 days)	
**Indicator Variables**	No	Yes	No	Yes
**Male**	Count (%)	Count (%)	Count (%)	Count (%)
Tobacco Smoking	2673 (75.2)	879 (24.8)	3366 (94.8)	186 (5.2)
Electronic (E) Cigarette Smoking	2609 (73.5)	943 (26.5)	3518 (99.0)	34 (1.0)
Alcohol Drinking	2038 (57.4)	1514 (42.6)	2886 (81.2)	666 (18.8)
Cannabis/Kratom-related Consumption	2459 (69.2)	1093 (30.8)	3158 (88.9)	394 (11.1)
Stimulant/opioid substances Consumption	3400 (95.7)	152 (4.3)	3523 (99.2)	29 (0.8)
Club/Party Drug Consumption	3366 (94.8)	186 (5.2)	3513 (98.9)	39 (1.1)
Non-Medical Pharmaceutical Drug Consumption	2713 (76.4)	839 (23.6)	3348 (94.3)	204 (5.7)

**Female**				
Tobacco Smoking	4526 (92.8)	354 (7.3)	4865 (99.7)	15 (0.3)
Electronic (E) Cigarette Smoking	4281 (87.7)	599 (12.3)	4873 (99.9)	7 (0.1)
Alcohol Drinking	2797 (57.3)	2083 (42.7)	4091 (83.8)	789 (16.2)
Cannabis/Kratom-related Consumption	4179 (85.6)	701 (14.4)	4692 (96.1)	188 (3.9)
Stimulant/opioid substances Consumption	4819 (98.7)	61 (1.3)	4874 (99.9)	6 (0.1)
Club/Party Drug Consumption	4774 (97.8)	106 (2.2)	4861 (99.6)	19 (0.4)
Non-Medical Pharmaceutical Drug Consumption	3780 (77.5)	1100 (22.5)	4588 (94.0)	292 (6.0)

**Gender Diverse**				
Tobacco Smoking	358 (88.6)	46 (11.4)	403 (99.7)	1 (0.3)
Electronic (E) Cigarette Smoking	341 (84.4)	63 (15.6)	404 (100)	–
Alcohol Drinking	191 (47.3)	213 (52.7)	332 (82.2)	72 (17.8)
Cannabis/Kratom-related Consumption	323 (80.0)	81 (20.0)	380 (94.1)	24 (5.9)
Stimulant/opioid substances Consumption	394 (97.5)	10 (2.5)	401 (99.3)	3 (0.7)
Club/Party Drug Consumption	391 (96.8)	13 (3.2)	399 (98.8)	5 (1.2)
Non-Medical Pharmaceutical Drug Consumption	312 (77.2)	92 (22.8)	370 (91.6)	34 (8.4)

⁎ Frequent exposure in the past 30 days in relation to tobacco and e-cigarette smoking refers to participants engaging in consumption of substances for ≥20 days within the past 30-day period.

**Table 2:** Model fit indices for Latent Class Models of Substance use 1 through 5 based on 5th National School Survey on Alcohol Consumption, Substance Use and Other Health-Risk Behaviors study participants aged 16–17 Thai Male, Female and Gender Diverse adolescents respectively in 2020/2021.

CLASS Model	Number of Observations	*P*-Value	Log-likelihood	AIC	BIC	ENTROPY
*Males*						
1	3552	< 0.01	−16,995.228	34,018.455	34,104.909	–
2	3552	< 0.01	−14,599.076	29,250.153	29,410.710	0.82171
3	3552	< 0.01	−13,991.521	28,057.042	28,285.527	0.848421
4	3552	< 0.01	−13,726.336	27,560.672	27,894.136	0.84735
5	3552	0.950	−13,613.137	27,362.273	27,782.191	0.842517

*Females*						
1	4880	< 0.01	−16,254.352	32,536.703	32,627.604	–
2	4880	< 0.01	−14,267.217	28,580.433	28,729.770	0.825206
3	4880	< 0.01	−13,612.522	27,309.044	27,581.746	0.827985
4	4880	0.357	−13,409.605	26,899.211	27,158.927	0.817344

*Gender Diverse*						
1	404	< 0.01	−1569.7389	3165.478	3217.496	–
2	404	< 0.01	−1404.9491	2853.898	2941.929	0.771135
3	404	< 0.01	−1329.4566	2722.913	2850.959	0.800459
4	404	0.028	−1290.4724	2680.945	2881.015	0.820709

Abbreviations: AIC, Akaike Information Criterion; BIC, Bayesian Information Criterion; −: entropy not computable. Boldface type indicates the selected model.

Most participants reported abstaining from substances: tobacco (85.6%), e-cigarettes (81.8%), stimulant/opioid substances (97.5%), and club/party drugs (96.5%). However, lifetime use was notable for alcohol (43.1%), non-medical pharmaceutical use (23.0%), cannabis/kratom-related substances (21.3%), e-cigarettes (18.2%), and tobacco (14.4%).

For frequent use in the past 30 days, alcohol had the highest prevalence (17.1%), followed by cannabis/kratom-related substances (6.9%) and non-medical pharmaceutical use (6.0%). Tobacco accounted for 2.2%, while other drugs were below 1%.

Gender-stratified analysis showed females (85–92%) and gender-diverse individuals (80–88%) were more likely to abstain from tobacco, e-cigarettes, and cannabis/kratom-related substances compared to males (69–73%). Males had over twice the prevalence of lifetime substance use, though lifetime alcohol use was similar across males (42.6%), females (42.7%), and gender-diverse individuals (52.7%). Frequent use was significantly higher among males.

A gender-stratified latent class analysis identified substance use patterns among adolescents aged 16–17. Model fit was evaluated using log-likelihood, AIC, BIC, and entropy (≥0.8) per best practices (27).

Subsequent model solutions were iteratively compared across fit statistics to identify the optimal number of classes. Based on AIC, BIC, log-likelihood, and entropy results, a four-class model for males and three-class models for females and gender-diverse participants were selected. These provided the best balance of model fit and parsimony, capturing the underlying structure and subgroups in the data (27).

Participants' posterior probability scores were calculated using the Bayesian approach (27) to assign class membership, with individuals placed in the class with the highest score. A new categorical variable was created for class membership. Table 3 shows estimated frequencies based on posterior probabilities, identifying four user-pattern classes for males and three for females and gender-diverse participants.

**Table 3:** Frequency and percentage of participants in each of the classes based on estimated posterior probability of class membership for each gender respectively.

CLASS	FREQUENCY (n)	PERCENTAGE (%)	Average Posterior Probabilities Matrix			
			Class I	Class II	Class III	Class IV
*Males*						
I: minimal/non-use	1982	55.8	0.950	0.025	0.024	0.000
II: experimental cannabis/kratom and tobacco-related use	338	9.5	0.079	0.814	0.083	0.024
III: predominantly alcohol-focused use	934	26.3	0.058	0.034	0.884	0.023
IV: high polysubstance use	298	8.4	0.001	0.017	0.054	0.929

*Females*						
I: minimal/non-use	3605	73.9	0.917	0.075	0.008	
II: alcohol-focused	765	15.7	0.018	0.930	0.053	
III: high polysubstance use	510	10.4	0.055	0.029	0.917	

*Gender Diverse*						
I: minimal/non-use	253	62.6	0.898	0.097	0.005	
II: alcohol-focused	103	25.5	0.053	0.925	0.022	
III: high polysubstance use	48	11.9	0.081	0.08	0.841	

Across all gender groups, the largest latent class consisted of minimal or non-users, while additional classes reflected alcohol-focused and broader polysubstance involvement. Among males, a four-class solution was retained because it provided greater differentiation of substance use behaviors relative to the three-class solution while maintaining interpretability and class stability. Among females and gender-diverse adolescents, three-class solutions were selected because additional classes produced limited substantive differentiation.

Male classes were characterized as: (I) minimal/non-use class, (II) experimental cannabis/kratom and tobacco-related use class, (III) predominantly alcohol-focused use class, and (IV) high polysubstance use class. Female and gender-diverse participants demonstrated similar three-class structures consisting of minimal/non-use, alcohol-focused, and high polysubstance use classes.

Across all genders, adolescents assigned to classes other than the minimal/non-use class demonstrated higher odds of screening positive for elevated depressive symptoms. Notably, among males, adolescents in the experimental cannabis/kratom and tobacco-related class demonstrated odds of screening positive for elevated depressive symptoms comparable to those observed among the high polysubstance use class.

**Fig. 1:**
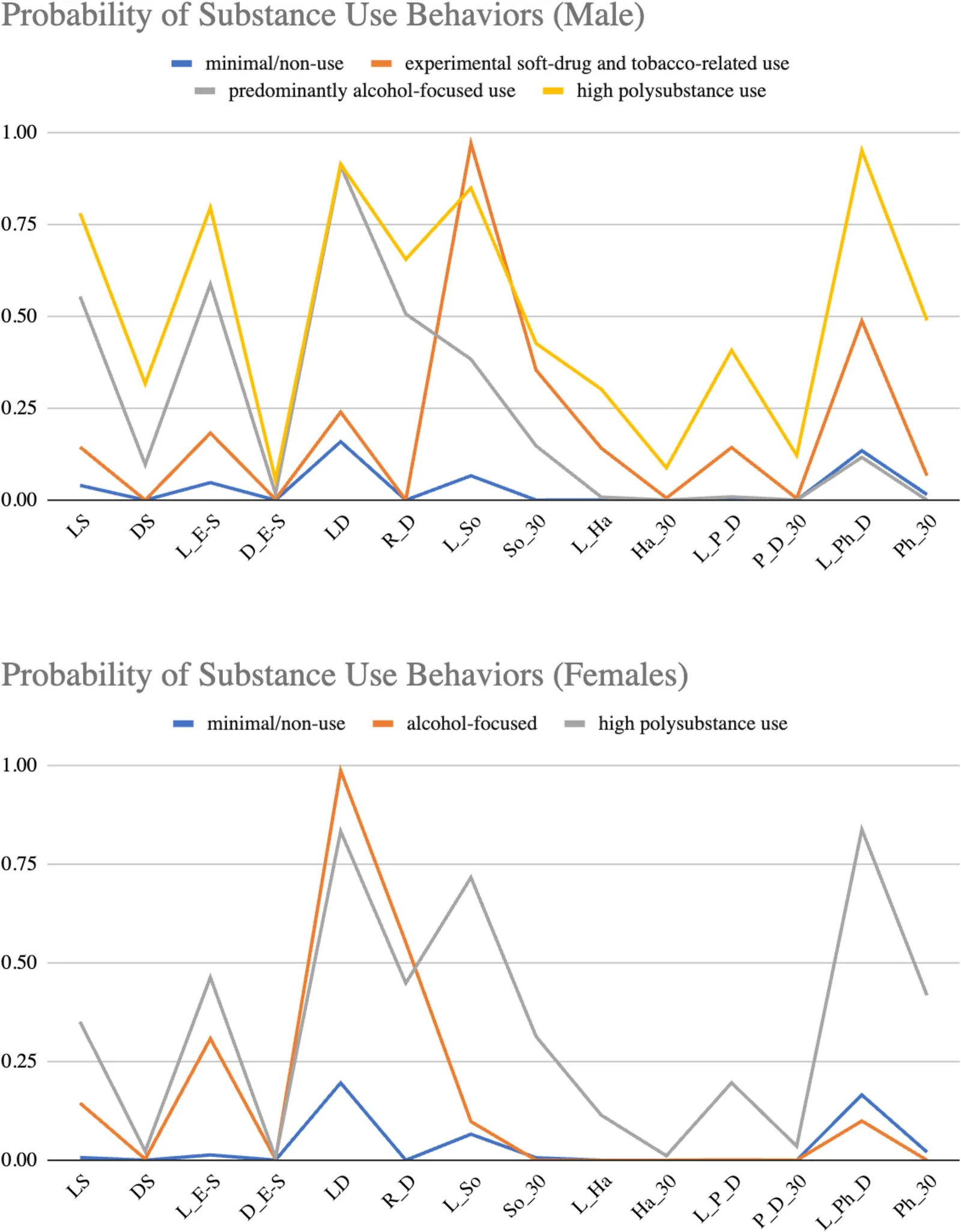

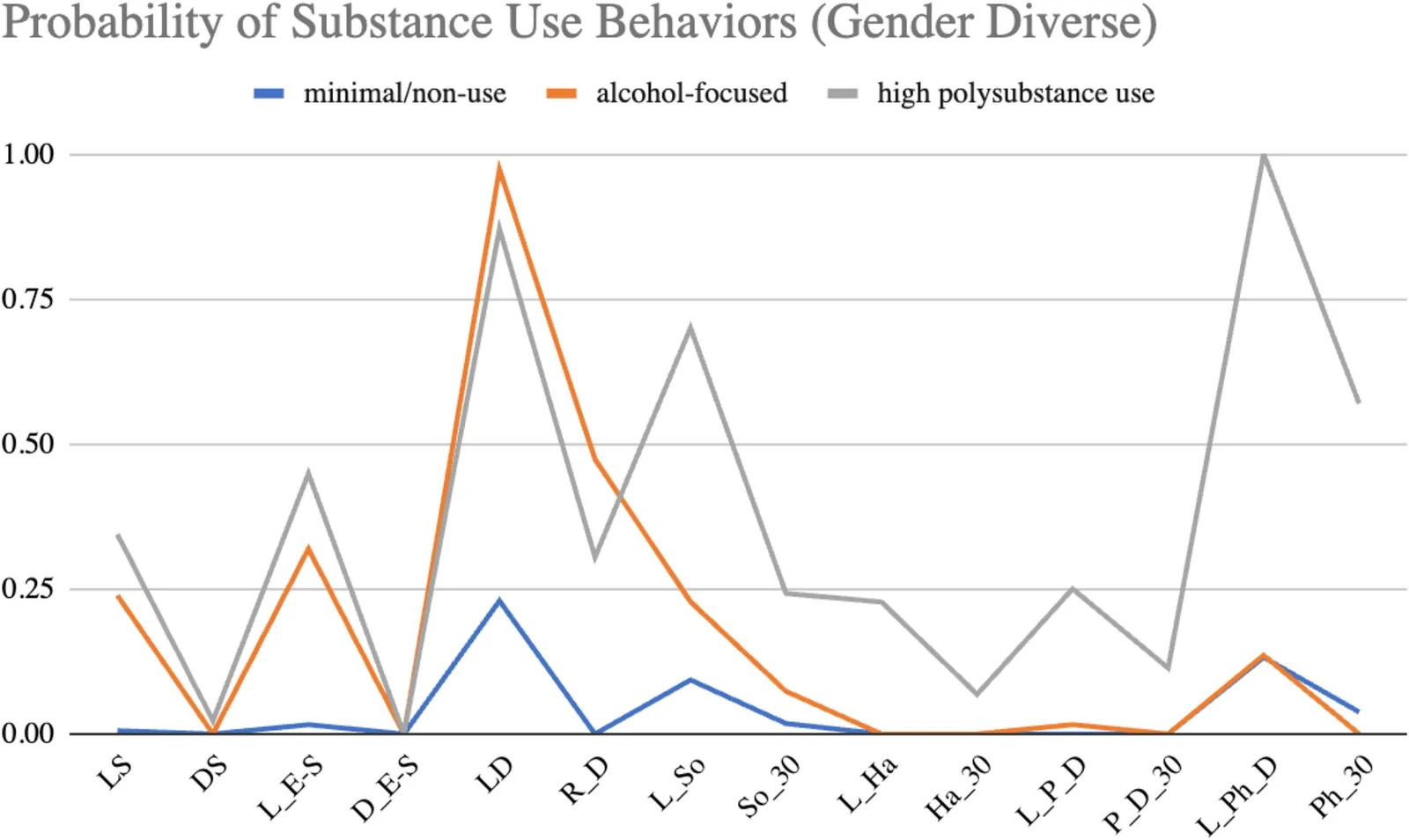
Gender stratified item probability of Substance use extracted from the data of 5th National School Survey on Alcohol Consumption, Substance Use and Other Health-Risk Behaviors study participants aged 16–17 in 2020/2021 (see legend below for abbreviations). *Abbreviations: LS: Lifetime Smoking; DS: Daily Smoking in the past 30 days; L_*E*-S: Lifetime E-cigarette smoking; D_E—S: Daily E-cigarette smoking in the past 30 days; LD: Lifetime Drinker; R_D: Indicated for Risky drinking based on Audit-C; L_So: Lifetime use of Soft Drugs; So_30: Use of soft drugs in the past 30 days; L_Ha: Lifetime use of Hard Drugs; Ha_30: Use of hard drugs in the past 30 days; L_P_D: Lifetime use of Party drugs; P_D_30: Use of party drugs in the past 30 days; L_Ph_D: Lifetime use of Pharmaceutical drugs; Ph_30: Use of pharmaceutical drugs in the past 30 days.

Fig. 1 illustrates item response probabilities by latent class marginal means. Following best practices, a threshold of >0.80 was used to categorize classes by substance type (Weller et al., 2020).

Among males in Class I, indicator variables showed minimal marginal means, indicating a non-user profile with some lifetime exposure to legal drugs. Class II was similar, except lifetime cannabis/kratom-related use exceeded 0.80 probability. In Class III, lifetime drinking was above 0.80, while frequent alcohol and cannabis/kratom-related use hovered around 0.5. Class IV had multiple items above 0.80, including lifetime smoking, e-cigarette use, drinking, cannabis/kratom-related substances, and non-medical pharmaceutical use. Overall, Class IV students scored higher across most items compared to Class I.

Females and gender-diverse participants showed similar patterns. Class I indicated non-users. Class II had lifetime drinking above 0.8 and frequent drinking around 0.6, with other items low. Class III exceeded 0.8 for lifetime drinking and showed high probabilities for lifetime cannabis/kratom-related and non-medical pharmaceutical drug use.

In the second part of the analysis, we assessed the correlation between latent classes and the risk of depressive symptoms (PHQ-2 ≥ 2 points). Table 4 shows the distribution of latent classes and covariates by gender. The likelihood of depressive symptoms was lowest in Class I across genders, while the highest risk occurred in Class IV for males and Class III for females and gender-diverse participants—groups with the most substance use.

**Table 4:** Association of latent classes and social demographic characteristics with being indicated with depressive symptoms (PHQ-2 ≥ 2 points).

Variable		Low PHQ Score, N (%)	High PHQ Score, N (%)	P-Value		Low PHQ Score, N (%)	High PHQ Score, N (%)	P-Value		Low PHQ Score, N (%)	High PHQ Score, N (%)	P-Value
*Gender*		*Male*				*Female*				*Gender Diverse*		
*Class*	I	1421 (71.7)	561 (28.3)			1845 (51.2)	1760 (48.8)	< 0.01		112 (44.3)	141 (55.7)	0.01
	II	197 (58.3)	141 (41.7)	< 0.01		293 (38.3)	472 (61.7)			30 (29.1)	73 (70.9)	
	III	604 (64.7)	330 (35.3)			168 (32.9)	342 (67.1)			13 (27.1)	35 (72.9)	
	IV	172 (57.7)	126 (42.3)									
*Covariates*												
*GPA Scores*	Up to 3.01	1241(68.9)	560 (31.1)	0.05		1277 (52.0)	1180 (48.0)	< 0.01		84 (41.2)	120 (58.8)	0.24
	More than 3.01	1153 (65.9)	598 (34.2)			1029 (42.5)	1394 (57.5)			71 (35.5)	129 (64.5)	
*Grades compared* *to previous year*	Same grades	449 (73.0)	166 (27.0)	< 0.01		314 (50.8)	304 (49.2)	< 0.01		27 (52.9)	24 (47.1)	< 0.01
	Better grades	1340 (68.8)	607 (31.2)			1389 (49.7)	1407 (50.3)			92 (43.2)	121 (56.8)	
	Worse grades	540 (60.9)	347 (39.1)			521 (40.6)	762 (59.4)			33 (26.6)	91 (73.4)	
	Missing	65	38			82	101			3	13	
*Weekly Allowance*	0–500 THB	1515 (66.5)	765 (33.5)	0.11		1648 (47.2)	1845 (52.8)	0.87		82 (34.5)	156 (65.5)	0.05
	501+ THB	879 (69.1)	393 (30.9)			658 (47.4)	729 (52.6)			73 (44.0)	93 (56.0)	
*Earned Income*	No	1373 (68.4)	633 (31.6)	0.22		1504 (48.9)	1570 (51.1)	< 0.01		90 (38.1)	146 (61.9)	0.38
	Yes	1011 (66.0)	522 (34.1)			799 (44.5)	995 (55.5)			65 (39.4)	100 (60.6)	
	Missing	10	3			3	9			0	3	
*Residence*	Home	2089 (68.8)	3036 (31.2)	< 0.01		2027 (48.4)	2161 (51.6)	< 0.01		123 (38.2)	199 (61.8)	0.78
	School	80 (60.2)	53 (39.8)			48 (39.7)	73 (60.3)			5 (41.7)	7 (58.3)	
	Others	209 (57.6)	154 (42.4)			221 (39.9)	333 (60.1)			25 (37.3)	42 (62.7)	
	Missing	16	4			10	7			2	1	
*Sleep, Mean (SD)*		6.62 (1.38)	6.2 (1.34)	< 0.01		6.60 (1.31)	6.16 (1.22)	< 0.01		6.24 (1.41)	5.76 (1.27)	< 0.01

Insufficient sleep was significantly associated with depressive symptoms, as high-risk students reported fewer hours of sleep. Other covariates—GPA, grade changes, weekly allowance, residence, sleep, and earned income—were statistically significant (*p* < 0.01) and included in logistic regression. Missing data were excluded.

Across genders, depressive symptoms were more common among students with GPA >3 and those reporting worse grades compared to the previous year. Students with same or better grades had lower risk. Socioeconomic factors showed mixed results: weekly allowance was not significant for males and females but was linked to higher risk among gender-diverse students. Earned income showed no difference for males and gender-diverse participants but was associated with higher risk in females. Not living at home increased risk for males and females.

Logistic regression examined associations between class membership and depressive symptoms, adjusting for significant covariates. Table 5 presents odds ratios (OR) and 95% confidence intervals for fully adjusted models.

**Table 5:** Gender stratified combined logistic regression analysis across all classes for the association between predicted probability of class membership based on substance use behaviour by likelihood of being indicated with depressive symptoms (PHQ-2 ≥ 2 points). Odds ratio (OR) and associated 95% confidence interval are presented below.

Variable		Males	Females	Gender Diverse
Class	I	1.0 (Ref.)	1.0 (Ref.)	1.0 (Ref.)
	II	1.64 (1.28–2.10)***	1.61 (1.36–1.91)***	1.96 (1.17–3.30)**
	III	1.32 (1.11–1.57)***	1.92 (1.57–2.36)***	2.13 (1.03–4.40)*
	IV	1.69 (1.30–2.20)***	n/a	n/a
GPA	Cont.	ns.	1.10 (1.01–1.10)**	ns.
Grades compared to previous year	Same grades	1.0 (Ref.)	1.0 (Ref.)	1.0 (Ref.)
	Better grades	1.23 (1.0–1.51)	1.09 (0.91–1.31)	1.48 (0.78–2.82)
	Worse grades	1.69 (1.34–2.13)***	1.50 (1.23–1.84)**	3.14 (1.54–6.39)***
Weekly Allowance	0–500 THB	ns.	ns.	1.0 (Ref.)
	501+ THB			0.61 (0.39–0.95)*
Earned Income	No	ns.	1.0 (Ref.)	ns.
	Yes		1.12 (0.99–1.27)	
Residence	Home	1.0 (Ref.)	1.0 (Ref.)	ns.
	School	1.61 (1.10–2.37)**	1.73 (1.17–2.56)**	
	Others	1.61 (1.28–2.03)***	1.35 (1.12–1.63)***	
Sleep	Cont.	0.81 (0.77–0.86)***	0.77 (0.73–0.80)***	0.74 (0.62–0.87)***

*p value is <0.05 **p value is <0.01 *** *p* value <0.001.

Abbreviations: Ref. = reference category; ns. = Not significantly associated with being indicated with depressive symptoms (PHQ-2≥) (see Table 4); Cont. = Continuous variable, testing for trend.

Among all students, belonging to a Class other than Class I, even after adjusting for confounders, was significantly associated with a higher risk of depressive symptoms. These findings suggest substance use correlates with poorer mental health. Notably, among male students, those in Class II (cannabis/kratom-related use) showed a similar risk of depressive symptoms as Class IV (polysubstance use), indicating that even less extensive drug use may lead to comparable mental health outcomes.

Among covariates, insufficient sleep, worse grades than the previous year, and living away from home were linked to higher risk of depressive symptoms among males. The same applied to females, with the addition that having a GPA >3.0 was also associated with higher risk. For gender-diverse students, shorter sleep, worse grades, and low weekly allowance significantly increased the risk of depressive symptoms.

Table 6 shows gender-stratified odds ratios for all covariates in each latent class regarding risk of depressive symptoms. Several covariates among Class I students, regardless of gender, were significantly associated with higher risk. Among males in Class IV (poly-substance use), insufficient sleep was linked to higher risk. In Class III (primarily alcohol users), low weekly allowance, boarding school living, and insufficient sleep were associated with depressive symptoms. Interestingly, insufficient sleep was not linked to higher risk among Class II males (cannabis/kratom-related users). Among females in Class III—the group with most prevalent substance use—insufficient sleep and undefined living arrangements were associated with higher risk. For both females and gender-diverse students, insufficient sleep significantly increased risk in Class II, indicating extensive alcohol use without other substances.

**Table 6:** Logistic regression analysis for the association between predicted probability of class Membership based on substance use behaviour across genders by likelihood of being indicated with depressive symptoms (PHQ-2 ≥ 2 points), stratified by class. Odds ratio (OR) and associated 95% confidence interval are presented below.

		Male				Female			Gender Diverse		
Variable		Class I	Class II	Class III	Class IV	Class I	Class II	Class III	Class I	Class II	Class III
		(*N* = 1982)	(*N* = 338)	(*N* = 934)	(*N* = 298)	(*N* = 3605)	(*N* = 765)	(*N* = 510)	(*N* = 253)	(*N* = 103)	(*N* = 48)
GPA Score	Cont.	1.01	1.02	1.01	1.04	1.11	1.05	1.01	0.98	1.79	0.90
		(0.94–1.08)	(0.89–1.15)	(0.93–5.30)	(0.90–1.20)	(1.05–1.17)	(0.95–1.16)	(0.93–5.30)	(0.79–1.23)	(0.84–4.07)	(0.51–1.59)
Grades compared to previous year	Same	1.0 (ref.)	1.0 (ref.)	1.0 (ref.)	1.0 (ref.)	1.0 (ref.)	1.0 (ref.)	1.0 (ref.)	1.0 (ref.)	1.0 (ref.)	1.0 (ref.)
	Better	1.07	2.08	1.19	1.79	1.04	1.09	1.64	2.23	0.18	1.10
		(0.80–1.43)	1.00–4.31)	(0.80–1.75)	(0.93–3.42)	(0.84–1.28)	(0.69–1.72)	(0.93–2.87)	(0.96–5.17)	(0.19–1.63)	(0.10–11.6)
	Worse	2.02***	2.08	1.39	1.64	1.50***	1.27	1.81	5.38***	0.24	8.09
		(1.31–2.47)	(0.95–4.54)	(0.89–2.14)	(0.81–3.34)	(1.19–1.90)	(0.77–2.09)	(0.98–3.35)	(2.14–13.50)	(0.02–2.39)	(0.49–33.1)
Weekly Allowance	0–500 THB	1.0 (ref.)	1.0 (ref.)	1.0 (ref.)	1.0 (ref.)	1.0 (ref.)	1.0 (ref.)	1.0 (ref.)	1.0 (ref.)	1.0 (ref.)	1.0 (ref.)
	501+ THB	0.75*	0.69	0.74*	1.32	0.87**	0.75	1.08	0.60	0.42	0.50
		(0.60–0.94)	(0.41–1.16)	(0.55–0.99)	(0.81–2.15)	(0.74–0.92)	(0.54–1.04)	(0.71–1.66)	(0.33–1.07)	(0.15–1.18)	(0.10–2.56)
Earned Income	No	1.0 (ref.)	1.0 (ref.)	1.0 (ref.)	1.0 (ref.)	1.0 (ref.)	1.0 (ref.)	1.0 (ref.)	1.0 (ref.)	1.0 (ref.)	1.0 (ref.)
	Yes	1.10	1.08	0.87	0.71	1.19**	0.76	1.29	0.99	1.08	0.22
		(0.89–1.37)	(0.68–1.72)	(0.66–1.15)	(0.43–1.16)	(1.03–1.38)	(0.54–1.03)	(0.87–1.93)	(0.54–1.78)	(0.68–1.72)	(0.04–1.14)
Residence	Home	1.0 (ref.)	1.0 (ref.)	1.0 (ref.)	1.0 (ref.)	1.0 (ref.)	1.0 (ref.)	1.0 (ref.)	1.0 (ref.)	1.0 (ref.)	1.0 (ref.)
	School	1.56	3.03**	3.87**	0.64	1.67**	2.27	n/a	0.39	n/a	n/a
		(0.92–2.64)	(1.78–7.81)	(1.42–10.6)	(0.16–2.62)	(1.10–2.54)	(0.61–8.51)		(0.06–2.71)		
	Other	2.02***	1.42	1.39	1.43	1.36**	1.23	1.82*	1.01	0.61	0.28
		(1.44–2.82)	(0.68–1.72)	(0.92–2.11)	(0.69–2.95)	(1.08–1.71)	(0.77–1.96)	(1.03–3.23)	(0.46–2.24)	(0.19–1.98)	(0.02–3.33)
Sleep	Cont.	0.82***	0.70	0.78***	0.82*	0.76***	0.79***	0.70***	0.71***	0.68*	1.43
		(0.76–0.89)	(0.68–1.72)	(0.71–0.88)	(0.69–0.98)	(0.72–0.81)	(0.70–0.89)	(0.60–0.82)	(0.56–0.89)	(0.48–0.96)	(0.75–2.73)

*p value is <0.05 **p value is <0.01 *** *p* value <0.001; Abbreviations: Ref. = reference category; n/a. = no analyzable cases; Cont. = Continuous variable, testing for trend.

## 4 Discussion

This study identified distinct gender-stratified substance use patterns among Thai adolescents and demonstrated that adolescents assigned to polysubstance-related classes generally had higher odds of screening positive for elevated depressive symptoms relative to minimal/non-users. The findings additionally suggest heterogeneity in substance use profiles across gender groups, particularly among males where a distinct experimental cannabis/kratom and tobacco-related class emerged.

Our findings align with prior research in other adolescent populations identifying a 4-class model (Delk et al., 2019; Gilreath et al., 2014; Göbel et al., 2016; Kulis et al., 2016; Park & Kim, 2017; Williams et al., 2021), where the largest class across all gender groups consisted of minimal or non-users, while additional classes reflected alcohol-centered and broader polysubstance involvement (Gilreath et al., 2014; Park & Kim, 2017; Williams et al., 2021), and e-cigarettes (Delk et al., 2019), with some studies including inhalants and illicit drugs (Göbel et al., 2016; Kulis et al., 2016). However, the present findings also demonstrated several context-specific differences. In particular, male adolescents exhibited a distinct experimental cannabis/kratom and tobacco-related class that was less evident among females and gender-diverse adolescents. These differences may reflect gendered social norms, differential access to substances, or variations in peer-group environments within the Thai context.

Most Thai adolescents transition into adulthood with low or moderate use of alcohol and drugs (Assanangkornchai et al., 2018) and previous longitudinal studies suggest that substance use patterns may remain relatively stable over time (Choi et al., 2018). The high polysubstance use class uniquely included adolescents reporting both lifetime and current cannabis/kratom-related use, aligning with Ozeylem et al. (2021), who found marijuana most prevalent among Thai adolescents.

Nearly 50% of substance users reported a positive likelihood of depressive symptoms, though the cross-sectional design limits causal inference. Only one similar study on Canadian adolescents found females and males in polysubstance classes had 2.65 and 1.69 higher odds of depression, respectively, versus non-users; dual-use classes showed odds of 1.48 and 1.21 (Williams et al., 2021). The relationship is complex and bidirectional—substance use may increase depression risk, while depressive symptoms can drive self-medication.

Gender identity was the most prominent factor. Females consistently showed higher risk across substance-using classes, aligning with Williams et al. (2021) and other studies reporting high co-occurrence of depression and substance use among adolescent women (Uma Rao & Shannon, 2000), and elevated odds for those initiating use before age 18 (Xu et al., 2016). A meta-analysis confirmed higher depression prevalence among Asian female adolescents (Shorey et al., 2021). Poulin et al. (2005) also found alcohol, smoking, and cannabis predicted depressive symptoms in females. Gender-diverse students were at significant risk, supported by research showing sexual minorities have higher substance use and double depression rates compared to heterosexual peers (Bhatia et al., 2023; Lucassen et al., 2017), though wide CIs may reflect small sample size.

While females and gender-diverse individuals dominate findings, males remain relevant. Studies in Texas and Malaysia show males more likely to engage in polysubstance use and remain in harmful patterns (Choi et al., 2018; Rodzlan Hasani et al., 2017). Socio-economic factors also matter. Boarding school or living away from home significantly increased depression risk, consistent with research linking non-family living to substance use, likely due to reduced parental monitoring (Dittus et al., 2023; Park & Kim, 2017) and peer influence (Hall et al., 2016; Nawi et al., 2021), however these mechanisms were not directly measured in the present study.

Longer sleep duration was associated with lower odds of screening positive for elevated depressive symptoms, consistent with broader adolescent mental health literature linking sleep disruption with emotional dysregulation, substance use, and depressive symptoms (Palmer & Alfano, 2017; Pasch et al., 2012). The relationship is likely bidirectional, as depressive symptoms may contribute to sleep disruption while insufficient sleep may increase emotional vulnerability and maladaptive coping behaviors.

### 4.1 Strengths

This study benefits from a large, well-represented sample of Thai adolescents aged 16–17, with a high response rate. Data covers school-attending students from both urban and rural areas, providing strong national representation. Findings offer preliminary evidence for future research and can inform policymakers on identifying high-risk students for substance use and depression, supporting targeted health promotion strategies.

### 4.2 Limitations

The cross-sectional design limits causal inference (Wang & Cheng, 2020). The observed associations between substance use classes and elevated depressive symptoms should be interpreted cautiously given the cross-sectional design. The findings indicate co-occurrence rather than causal directionality. Substance use patterns may reflect broader psychosocial vulnerability, behavioral dysregulation, self-medication processes, or shared environmental adversity (Khantzian, 1997; Thatcher & Clark, 2008).

Self-reported data introduces potential bias, including social desirability, as sensitive questions on mental health and drug use may lead participants to modify responses (Wang & Cheng, 2020). Risk stratification was not possible since categories included psychostimulants, which may cause mania rather than depression. Excluding out-of-school youth reduces generalizability. Data collected in early 2021 may reflect COVID-19 conditions, limiting broader applicability; some vulnerable students may have missed school (Pal et al., 2022), and return rates are unknown. Thus, results may not fully capture substance use prevalence.

## 5 Conclusion

In this national sample of Thai adolescents aged 16–17 years, distinct gender-stratified substance use patterns were identified, with polysubstance-related classes generally showing higher odds of screening positive for elevated depressive symptoms relative to minimal/non-users. The findings contribute to the limited evidence on adolescent substance use heterogeneity within Southeast Asia and suggest that latent class patterns of substance use may be relevant for understanding adolescent mental health vulnerability. Longitudinal research is needed to clarify temporal relationships between substance use patterns and depressive symptoms.

## Ethical approval and data transparency

The present study is based on secondary analysis of anonymised, publicly available data obtained from the World Health Organization (WHO) NCD Microdata Repository. Ethical approval for the original data collection was granted by the Human Research Ethics Committee, Faculty of Medicine, Prince of Songkla University. The authors did not have access to any identifiable personal information.

## CRediT authorship contribution statement

**Renarebecca Sagayanathan:** Writing – original draft, Visualization, Methodology, Formal analysis, Conceptualization. **Wit Wichaidit:** Writing – review & editing, Validation, Methodology, Conceptualization. **Martin Stafström:** Writing – review & editing, Supervision, Funding acquisition, Conceptualization.

## Funding

This research received no specific grant from any funding agency in the public, commercial, or not-for-profit sectors. Renarebecca Sagayanathan received a scholarship from the Faculty of Medicine, Lund University. Martin Stafström contributed funding to this scholarship through his institutional research funds. The funding sources had no role in the study design, data collection, analysis, interpretation of results, or manuscript preparation.

## Declaration of competing interest

The authors declare that they have no known competing financial interests or personal relationships that could have appeared to influence the work reported in this paper.

## Data Availability

The data is publicly available from the WHO MIcrodata repository
