# Supplementary material for: Association between latent classes of tobacco, e-cigarette, alcohol, and illicit drug use patterns and depression among Thai adolescents
Source: Addict Behav Rep. 2026 Aug 26;24:100738. doi: 10.1016/j.abrep.2026.100738 (PMC13571557; doi:10.1016/j.abrep.2026.100738)
Supplement: Supplementary file 1 [file mmc1.docx]

Supplement - **Mean posterior probabilities**

**Males**

| Variable | Abbreviation in Figure 1 | Class: minimal/non-use | Class: experimental soft-drug and tobacco-related use | Class: predominantly alcohol-focused use | Class: high polysubstance use |
| --- | --- | --- | --- | --- | --- |
| Lifetime Smoking | LS | 0.04 | 0.14 | 0.55 | 0.78 |
| Daily Smoking in the past 30 days | DS | 0.00 | 0.00 | 0.10 | 0.32 |
| Lifetime E-cigarette smoking | L_E-S | 0.05 | 0.18 | 0.59 | 0.80 |
| Daily E-cigarette smoking in the past 30 days | D_E-S | 0.00 | 0.00 | 0.02 | 0.06 |
| Lifetime Drinker | LD | 0.16 | 0.24 | 0.91 | 0.91 |
| Indicated for Risky drinking based on Audit-C | R_D | 0.00 | 0.00 | 0.51 | 0.65 |
| Lifetime use of Soft Drugs | L_So | 0.07 | 0.97 | 0.38 | 0.85 |
| Use of soft drugs in the past 30 days | So_30 | 0.00 | 0.35 | 0.15 | 0.43 |
| Lifetime use of Hard Drugs | L_Ha | 0.00 | 0.14 | 0.01 | 0.30 |
| Use of hard drugs in the past 30 days | Ha_30 | 0.00 | 0.01 | 0.00 | 0.09 |
| Lifetime use of Party drugs | L_P_D | 0.00 | 0.14 | 0.01 | 0.41 |
| Use of party drugs in the past 30 days | P_D_30 | 0.00 | 0.00 | 0.00 | 0.12 |
| Lifetime use of Pharmaceutical drugs | L_Ph_D | 0.13 | 0.49 | 0.12 | 0.95 |
| Use of pharmaceutical drugs in the past 30 days | Ph_30 | 0.02 | 0.07 | 0.00 | 0.49 |

**Females**

| Variable | Abbreviation in Figure 1 | Class: minimal/non-use | Class: alcohol-focused | Class: high polysubstance use |
| --- | --- | --- | --- | --- |
| Lifetime Smoking | LS | 0.01 | 0.14 | 0.35 |
| Daily Smoking in the past 30 days | DS | 0.00 | 0.00 | 0.02 |
| Lifetime E-cigarette smoking | L_E-S | 0.01 | 0.31 | 0.46 |
| Daily E-cigarette smoking in the past 30 days | D_E-S | 0.00 | 0.00 | 0.01 |
| Lifetime Drinker | LD | 0.20 | 0.99 | 0.83 |
| Indicated for Risky drinking based on Audit-C | R_D | 0.00 | 0.55 | 0.45 |
| Lifetime use of Soft Drugs | L_So | 0.07 | 0.10 | 0.72 |
| Use of soft drugs in the past 30 days | So_30 | 0.01 | 0.00 | 0.31 |
| Lifetime use of Hard Drugs | L_Ha | 0.00 | 0.00 | 0.11 |
| Use of hard drugs in the past 30 days | Ha_30 | 0.00 | 0.00 | 0.01 |
| Lifetime use of Party drugs | L_P_D | 0.00 | 0.00 | 0.20 |
| Use of party drugs in the past 30 days | P_D_30 | 0.00 | 0.00 | 0.04 |
| Lifetime use of Pharmaceutical drugs | L_Ph_D | 0.17 | 0.10 | 0.84 |
| Use of pharmaceutical drugs in the past 30 days | Ph_30 | 0.02 | 0.00 | 0.42 |

**Gender diverse**

| Variable | Abbreviation in Figure 1 | Class: minimal/non-use | Class: alcohol-focused | Class: high polysubstance use |
| --- | --- | --- | --- | --- |
| Lifetime Smoking | LS | 0.01 | 0.24 | 0.34 |
| Daily Smoking in the past 30 days | DS | 0.00 | 0.00 | 0.02 |
| Lifetime E-cigarette smoking | L_E-S | 0.02 | 0.32 | 0.45 |
| Daily E-cigarette smoking in the past 30 days | D_E-S | 0.00 | 0.00 | 0.00 |
| Lifetime Drinker | LD | 0.23 | 0.97 | 0.87 |
| Indicated for Risky drinking based on Audit-C | R_D | 0.00 | 0.47 | 0.31 |
| Lifetime use of Soft Drugs | L_So | 0.09 | 0.23 | 0.70 |
| Use of soft drugs in the past 30 days | So_30 | 0.02 | 0.07 | 0.24 |
| Lifetime use of Hard Drugs | L_Ha | 0.00 | 0.00 | 0.23 |
| Use of hard drugs in the past 30 days | Ha_30 | 0.00 | 0.00 | 0.07 |
| Lifetime use of Party drugs | L_P_D | 0.00 | 0.02 | 0.25 |
| Use of party drugs in the past 30 days | P_D_30 | 0.00 | 0.00 | 0.11 |
| Lifetime use of Pharmaceutical drugs | L_Ph_D | 0.13 | 0.14 | 1.00 |
| Use of pharmaceutical drugs in the past 30 days | Ph_30 | 0.04 | 0.00 | 0.57 |
